# Design of New Genome- and Gene-Sourced Primers and Identification of QTL for Seed Oil Content in a Specially High-Oil *Brassica napus* Cultivar

**DOI:** 10.1371/journal.pone.0047037

**Published:** 2012-10-12

**Authors:** Meiyu Sun, Wei Hua, Jing Liu, Shunmou Huang, Xinfa Wang, Guihua Liu, Hanzhong Wang

**Affiliations:** Oil Crops Research Institute of the Chinese Academy of Agricultural Sciences, Key Laboratory of Biology and Genetic Improvement of Oil Crops, Ministry of Agriculture, Wuhan, People's Republic of China; Nanjing Agricultural University, China

## Abstract

Rapeseed (*Brassica napus* L.) is one of most important oilseed crops in the world. There are now various rapeseed cultivars in nature that differ in their seed oil content because they vary in oil-content alleles and there are high-oil alleles among the high-oil rapeseed cultivars. For these experiments, we generated doubled haploid (DH) lines derived from the cross between the specially high-oil cultivar zy036 whose seed oil content is approximately 50% and the specially low-oil cultivar 51070 whose seed oil content is approximately 36%. First, to address the deficiency in polymorphic markers, we designed 5944 pairs of newly developed genome-sourced primers and 443 pairs of newly developed primers related to oil-content genes to complement the 2244 pairs of publicly available primers. Second, we constructed a new DH genetic linkage map using 527 molecular markers, consisting of 181 publicly available markers, 298 newly developed genome-sourced markers and 48 newly developed markers related to oil-content genes. The map contained 19 linkage groups, covering a total length of 2,265.54 cM with an average distance between markers of 4.30 cM. Third, we identified quantitative trait loci (QTL) for seed oil content using field data collected at three sites over 3 years, and found a total of 12 QTL. Of the 12 QTL associated with seed oil content identified, 9 were high-oil QTL which derived from the specially high-oil cultivar zy036. Two high-oil QTL on chromosomes A2 and C9 co-localized in two out of three trials. By QTL mapping for seed oil content, we found four candidate genes for seed oil content related to four gene markers: GSNP39, GSSR161, GIFLP106 and GIFLP046. This information will be useful for cloning functional genes correlated with seed oil content in the future.

## Introduction

Rapeseed (*Brassica napus*) is one of the most important oilseed crops, and it is cultivated over a large area around the world. A 1% increase in the seed oil content of rapeseed is equivalent to a 2.3–2.5% increase in seed yield [1]. The vegetable oil in rapeseed is an important edible product and its value as an industrial resource is increasing, particularly as a resource for biodiesel production [2]. Therefore, it is very important to improve the seed oil content in rapeseed.

The detection of DNA sequence variation is of great importance in genetic studies of the *Brassica* genomes. Grodzicker et al. [3] initially introduced the restriction fragment length polymorphism (RFLP), and Botstein et al. [4] first proposed to use RFLP as genetic markers in genetic mapping, which pioneered the use of molecular markers as genetic markers. After that based on polymerase chain reaction (PCR), dozens of DNA molecular markers techniques such as random amplified polymorphic DNA (RAPD), amplified fragment length polymorphism (AFLP), cleaved amplified polymorphism sequences (CAPS), and simple sequence repeat (SSR) were successively developed. Along with the development of structural genomics and functional genomics, functional markers such as expressed sequences tag (EST), intron fragment length polymorphism (IFLP), target region amplification polymorphism (TRAP), single nucleotide polymorphism (SNP) and insertion-deletion (InDel) were developed based on the target gene which would be thought as an important development in the biological research. RFLP has been extensively used to compare mapping of the *Brassica* genomes [5] and also for comparative evolution in the related model plant *Arabidopsis thaliana* [6]. SSR, SNP and InDel have been extensively used in constructing genetic linkage map, mapping QTL, tagging qualitative genes, analyzing heterosis and dissecting the genetic basis of the *Brassica* genomes [7]–[18]. IFLP, SNP, and InDel have also been used in constructing genetic linkage map and mapping QTL in the other oil or cereal crops such as sunflower and Tef [19], [20].

Seed oil content of *B. napus* is a complicated quantitative trait controlled by multiple genes and also affected by the environment. In the past 20 years, many genetically separate populations have been created using different *B. napus* cultivars to identify quantitative trait loci (QTL) for seed oil content. In previous studies, the number of QTL for seed oil content in *B. napus* identified in 17 linkage groups (A1, A2, A3, A4, A5, A6, A7, A8, A9, A10, C1, C2, C3, C5, C6, C7, and C8) ranged from 3 to 27 [12], [16], [21]–[24]. The parents of the *B. napus* populations differed among those studies, and each parent had its own excellent genetic characteristics. However, there were only small differences among the parents of those *B. napus* populations, and few QTL associated with large phenotypic variations in seed oil content and with large additive effects were detected. Even fewer of those QTL were actually used in rapeseed breeding. Seed oil content is also influenced by the environment (temperature, rainfall, and light) [25]–[26]. The QTL for seed oil content detected in previous studies are difficult to repeat. In addition, the positions of QTL differ among various *B. napus* genomes [16], [22]. These findings suggest that there are different alleles for oil content among various *B. napus* cultivars. The restricted origins and history of selective breeding formed various rapeseed cultivars in nature with dramatic changes in the seed oil content. Meanwhile, different alleles containing candidate genes existed among various rapeseed cultivars.

To improve the seed oil content of rapeseed cultivars, it is useful to clone candidate genes correlated with seed oil content and use them in breeding programs. There are many difficulties in screening and identifying candidate genes for seed oil content in studies on detecting QTL for seed oil content in rapeseed. Also, it is time-consuming and laborious to finely map QTL for seed oil content. So far, only a few orthologous genes related to the lipid biosynthesis pathway in *A. thaliana* have been screened and identified from rapeseed [16], [27]. It was introduced that the fatty acid desaturase 2 (*FAD2*) gene was in the same QTL region in the *B. napus* DH population [27]. Then 14 orthologous genes involved in lipid synthesis pathway in *A. thaliana* were mapped to six QTL regions in a *B. napus* DH population [16]. These orthologous genes may be good candidate genes for seed oil content. There have been a few reports on combining mapping of candidate genes related to seed oil content with genetic linkage maps constructed in QTL analysis to screen and identify candidate genes for seed oil content [16], [27]. However, most of these publicly known functional candidate genes related to the lipid synthesis pathway were found in *A. thaliana*. There is a lack of specific candidate genes for seed oil content that are directly derived from rapeseed, since *B. napus* holds a much larger and more complicated genome than *A. thaliana*. Although many candidate genes were revealed by differential gene expression analysis for seed oil content in some rapeseed cultivars [28], [29], it was still difficult to identify those genes.

We have developed a specially high-oil cultivar zy036 whose seed oil content is approximately 50% [29], [30]. It may contain excellent alleles for high oil content. The special cultivar zy036 is the allotetraploid *B. napus* which combines the whole genome sequences of two diploid species *Brassica rapa* and *Brassica oleracea*. In our study, we aimed to (1) develop new genome-sourced primers based on the three genome sequences of *B. rapa*, *B. oleracea*, and *B. napus*; (2) develop new primers related to oil-content genes according to differentially expressed genes between two pools of individuals in the DH population showing extreme phenotypic variations in seed oil content; (3) construct a genetic linkage map and identify QTL for seed oil content in the specially high-oil cultivar zy036; (4) find molecular markers linked to seed oil content in the QTL; and (5) localize target candidate genes on the genetic linkage map to identify functional genes associated with seed oil content.

## Results

### Phenotypic Variation in Seed Oil Content in Parents and DH Population

**Figure 1:**
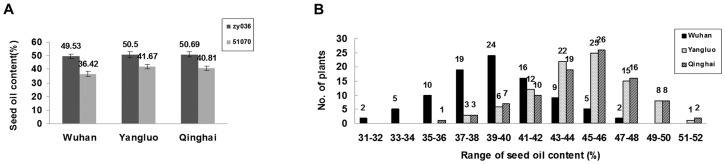
Phenotypic variation in seed oil content in the parents and DH population. **A. Distribution of average seed oil content ± SE in the parents zy036 and 51070.** Values in figure are average seed oil content (%) in the high-oil parent zy036 and low-oil parent 51070 in Wuhan (2010), Yangluo (2011), and Qinghai (2011) trials. **B. Frequency distribution of phenotypic variability in seed oil content (%) in the DH population.** Numbers in figure show number of plants in the DH population in Wuhan (2010), Yangluo (2011), and Qinghai (2011). In these three environments, seed oil content showed approximately continuous variations and normal distribution patterns.

Average values for seed oil content (± SE) in the high oil content parent zy036 and the low oil content parent 51070 in Wuhan (2010), Yangluo (2011), and Qinghai (2011) are summarized in Figure 1A. In the field trials in Wuhan, Yangluo, and Qinghai, zy036 (high oil content) showed an average seed oil content (± SE) of 49.53±1.73%, 50.5±2.22%, and 50.69±1.94%, respectively. There were no significant differences in average seed oil content among the three trials. In the same field trials, 51070 (low oil content) showed an average seed oil content (± SE) of 36.42±1.93%, 41.67±1.70%, and 40.87±1.60%, respectively. For this parental line, the average seed oil content was significantly lower (4–5%) in the field trial in Wuhan than in those in Yangluo or Qinghai using T-test (P<0.05).

The frequency distributions of seed oil content in the DH population in Wuhan (2010), Yangluo (2011), and Qinghai (2011) are summarized in Figure 1B. The average seed oil content of the DH population grown in Wuhan, Yangluo, and Qinghai ranged from 30.85 to 47.70%, 36.97 to 51.15%, and 36.20 to 51.30%, respectively. The seed oil content in the three environments showed approximately continuous variations and normal distribution patterns, as validated by the PROC NORMAL procedure (SAS 8.1, SAS INSTITUTE INC., USA). These observations also indicated that seed oil content in the parents and the DH population were lower in the trial in Wuhan than in those in Yangluo and Qinghai. This might caused by the environmental conditions in Wuhan, for example, higher rainfall or higher noon temperatures compared with those in Yangluo or Qinghai.

Using field phenotypic data of seed oil content in the DH population, we calculated Pearson coefficients using the PROC CORR procedure (SAS 8.1) to determine correlations among the three trials in Wuhan, Yangluo, and Qinghai. There were positive correlations among the three trials, as summarized in Table 1. The correlation between seed oil content in the Yangluo and Qinghai trials (r = 0.6112, P<0.0001) was higher than that between seed oil content in the Wuhan and Yangluo trials (r = 0.5145, P<0.0001) or that between seed oil content in the Wuhan and Qinghai trials (r = 0.3873, P = 0.0002).

**Table 1:** Correlations between seed oil content in three trials of DH population.

	oil1×oil2	oil1×oil3	oil2×oil3
r	0.5145	0.3873	0.6112
P	<0.0001	0.0002	<0.0001

*oil1* refers to seed oil content in Wuhan (2010).

*oil2* refers to seed oil content in Yangluo (2011).

*oil3* refers to seed oil content in Qinghai (2011).

### Design of Primers

Primer classifications are summarized in Table 2. In total, we designed 8631 pairs of primers including 2244 pairs of publicly available primers, 5944 pairs of newly developed genome-sourced primers, and 443 pairs newly developed primers related to oil-content genes.

**Table 2:** Primer classification, polymorphism screening in parents, and amplification in DH population.

Classification^a^	Primers prefix^b^	Type^c^	No.^d^	Polymorphism^e^	Amplification^f^	Markers^g^
	BN	SSR	24	2	1	2
	BnEMS, BnGMS, BoGMS, BrGMS	SSR	678	112	66	74
	BRAS, CB	SSR	310	40	21	24
	CNU	SSR	187	58	27	32
	EJU,ENA	SSR	25	5	3	3
	FITO	SSR	231	26	15	16
Public	IGF	SSR	95	11	4	4
	MR	SSR	23	5	2	3
	Na,Ni,Ol,Ra	SSR	398	59	30	34
	Niab	SSR	144	48	22	24
	sN, sR, sS	SSR	129	34	15	18
	Subtotal		2244	400	206	234
	BrSF	SSR	893	213	81	104
	BrBAC, P	SSR	378	91	53	63
	BoSF, SF	SSR	2422	289	169	181
Genome	Snap	SNP	2086	101	40	40
	Pr	SNP	71	9	5	5
	Ns	SNP	94	15	3	3
	Subtotal		5944	718	351	396
	GSSR	SSR	180	41	34	35
	GIFLP	IFLP	107	11	8	9
Gene	GSNP	SNP	42	10	6	6
	Gindel,GTFzip	INDEL	114	11	8	9
	Subtotal		443	73	56	59
	Total		8631	1191	613	689

a Primer classifications: *Public* indicates publicly available primers, *Genome* indicates newly developed genome-sourced primers, *Gene* indicates newly developed primers related to oil-content genes.

b Prefix for each primer when it was designed.

c Primer type based on its design.

d Total number of primers.

e Total number of polymorphic primers screened in parents zy036 and 51070.

f Total number of polymorphisms amplified by primers in DH population.

g Total number of molecular markers used to construct genetic linkage map in DH population.

Based on the results of the transcriptome data from two pools of individuals in the DH population with high and low seed oil contents, many differentially expressed genes were found (unpublished data). We chose 50 differentially expressed genes (Table S1) which showed obvious high-/low- expression between two pools to design gene primers. In the end, 443 pairs of newly developed primers related to oil-content genes were designed. These primers included 180 SSR, 107 IFLP, 42 SNP, and 114 InDel primers.

In addition, based on the results of the genome sequences of *B. rapa*, *B. oleracea*, and *B. napus*, we designed 5944 pairs of genome-sourced primers to amplify SSRs and SNPs in our laboratory. These primers included 3693 SSR and 2251 SNP primers.

### Primer Polymorphism Screening in the Parents

Primer screened for polymorphisms in the parents zy036 and 51070 are summarized in Table 2. A total of 8631 pairs of primers were screened to detect polymorphisms in the parents. There were 7588 (87.92%) pairs of primers that successfully amplified at least one clear band. Among the 7588 pairs of primers, most amplified one or two strong bands that corresponded to product(s) from the A genome, the C genome, or both. Only a few primer pairs amplified three to five bands.

As shown in Table 2, for the 2244 pairs of publicly available primers, 400(17.83%) amplified polymorphisms in the parents. For the 5944 pairs of newly developed genome-sourced primers, 718 (12.08%) amplified polymorphisms, and for the 443 pairs of newly developed primers related to oil-content genes, 73 (16.48%) amplified polymorphisms. Among the 5944 pairs of newly developed genome-sourced primers, 593 (16.06%) of the SSR and 125 (5.55%) of the SNP primers amplified polymorphisms in the parents. Among the 443 pairs of newly developed primers related to oil-content genes, 41 (22.78%) of the SSR, 11 (10.28%) of the IFLP, 10 (23.81%) of the SNP, and 11 (9.65%) of the InDel primers amplified polymorphisms in the parents.

The 1191 pairs of primers that revealed polymorphisms in the parents included 1034 SSR (86.82%), 11 IFLP (0.92%), 135 SNP (11.34%), and 11 InDel (0.92%) primers. Finally, the primers that amplified clear and simple bands were selected from the above 1191 primers to scan the DH population.

### Construction of Genetic Linkage Map

There were 613 pairs of primers including 543 SSR, 8 IFLP, 54 SNP and 8 InDel primers that amplified markers in the DH population (Table 2). We used 689 markers from the 613 pairs of primers to construct the genetic linkage map using Joinmap3.0. In the end, a framework of the genetic linkage map was constructed with 527 markers.

**Figure 2:**
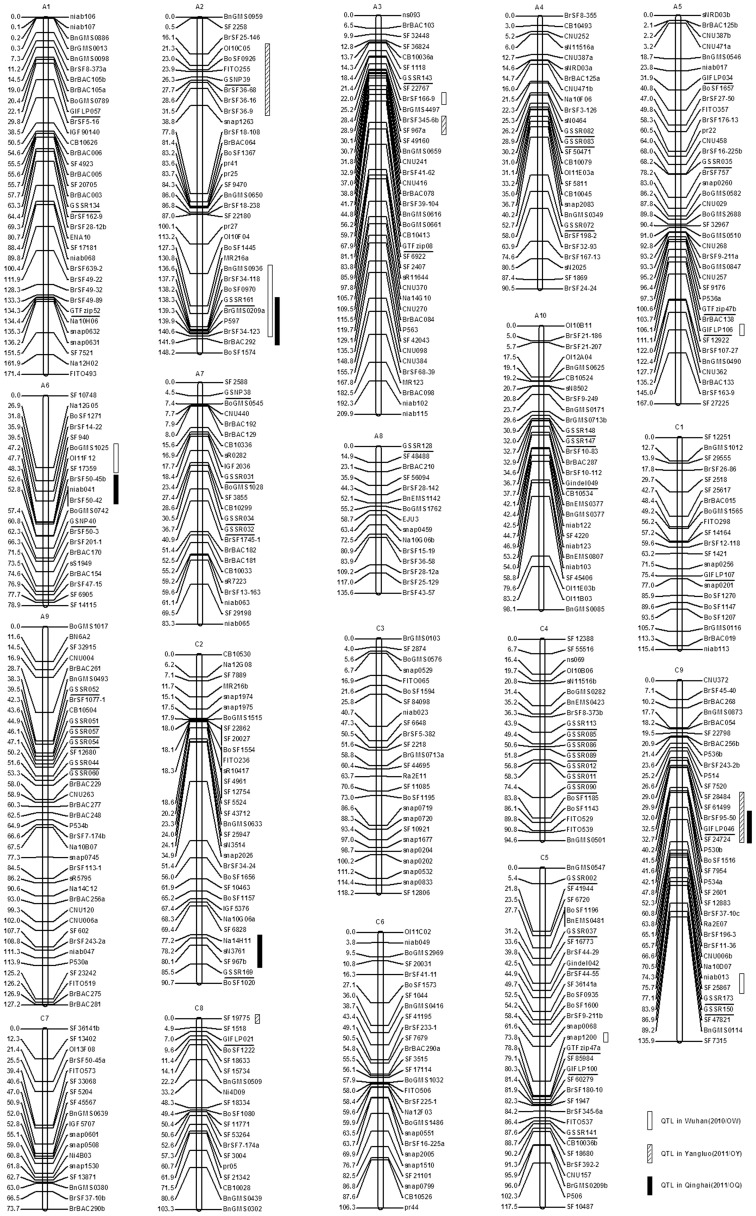
Genetic linkage map and positions of QTL in DH genetic linkage map. DH population was derived from a cross between zy036 and 51070. Linkage groups successfully assigned to A genome (A1–A10) and C genome (C1–C9). Newly developed primers related to oil-content genes are underlined and confidence intervals of QTL for seed oil content are tagged on the right side of linkage groups. White rectangles indicate QTL in Wuhan (2010/OW), striped rectangles indicate QTL in Yangluo (2011/OY), and black rectangles indicate QTL in Qinghai (2011/OQ).

The map (Figure 2, Table 3) included 181 publicly available markers, 298 newly developed genome-sourced markers (Table S2) and 48 newly developed markers related to oil-content genes (Table S3) using our DH population. The number of markers on each linkage map ranged from 15 (A8) to 39 (A3) with an average of 27.74 markers. Using the 181 public markers as the anchored markers, the 19 linkage groups (LGs) were successfully assigned to the 10 LGs in the A genome (A1–A10) and the 9 LGs in the C genome (C1–C9) following internationally accepted guidelines on nomenclature of *Brassica* genome LGs (http://www.brassica.info/resource/maps/lg-assignments.php), corresponding to the LGs N1–N10 and N11–N19 [31].

**Table 3:** Distribution of 527 markers on 19 linkage groups in DH map.

Linkage	Number of markers				Length	Density	No. of markers skewed to				
group	Public^a^	Genome^b^	Gene^c^	Total			zy036	51070	Pχ^2^	Total	Ratio(%)
A1	13	19	3	35	171.40	4.90	3	0	0.0833	3	8.57
A2	8	23	2	33	148.19	4.49	2	6	0.1573	8	24.24
A3	17	20	2	39	209.92	5.38	1	16	0.0003	17	43.59
A4	13	11	3	27	90.47	3.35	0	12	0.0005	12	44.44
A5	16	18	4	38	167.03	4.40	6	14	0.0736	20	52.63
A6	6	14	1	21	78.93	3.76	0	15	0.0001	15	71.43
A7	11	9	4	24	83.29	3.47	1	0	0.3173	1	4.17
A8	4	10	1	15	135.59	9.04	1	0	0.3173	1	6.67
A9	13	18	6	37	127.18	3.44	3	6	0.3173	9	24.32
A10	17	8	3	28	98.11	3.50	0	6	0.0143	6	21.43
C1	5	15	1	21	115.37	5.49	0	10	0.0016	10	47.62
C2	12	19	1	32	90.68	2.83	11	2	0.0126	13	40.63
C3	6	19	0	25	118.20	4.73	7	0	0.0082	7	28.00
C4	7	6	7	20	94.58	4.73	6	0	0.0143	6	30.00
C5	6	21	6	33	117.49	3.56	0	19	0.0000	19	57.58
C6	9	18	0	27	106.27	3.94	9	3	0.0833	12	44.44
C7	6	12	0	18	73.70	4.09	6	2	0.1573	8	44.44
C8	5	13	1	19	103.33	5.44	1	4	0.1797	5	26.32
C9	7	25	3	35	135.85	3.88	7	7	1.0000	14	40.00
Total	181	298	48	527	2265.54	4.30	64	122	0.0000	186	35.29

a *Public* indicates publicly available markers.

b *Genome* indicates newly developed genome-sourced markers.

c *Gene* indicates newly developed markers related to oil-content genes.

The total length of the 19 linkage groups was 2,265.54 cM, with an average length of 119.34 cM for each linkage group and an average distance of 4.30 cM between markers. The length of each linkage group ranged from 73.70 cM (C7) to 209.92 cM (A3) and the marker density in each linkage group ranged from 2.83 cM (C2) to 9.04 cM (A8).

The number of public markers on each linkage map ranged from 4 (A8) to 17 (A3) with an average of 9.53, that of newly developed genome-sourced markers ranged from 6 (C4) to 25 (C9) with an average of 15.68, and that of newly developed markers related to oil-content genes ranged from 0 (C3, C6, and C7) to 7 (C4) with an average of 2.53.

A total of 186 (35.29%) markers showed significant distortion of segregation (P<0.01) using the chi-square test, of which 64 (34.41%) skewed to zy036 and the other 122 (65.59%) skewed to 51070 (Table 3). These distorted markers affected almost all linkage groups. The number of distorted markers ranged from 1 (A7 and A8) to 20 (A5) and the ratio of distorted markers ranged from 4.17% (A7) to 71.43% (A6) for each linkage group. The majority of the distorted markers (65.59%) biased in favor of 51070. The distorted markers on C3 skewed mainly to zy036, and those on A3, A4, A6, C1, and C5 skewed mainly to 51070.

### QTL Mapping for Seed Oil Content

QTL mapping for seed oil content was performed at a LOD threshold of 3.54 in Wuhan (2010), 2.93 in Yangluo (2011), and 2.05 in Qinghai (2011) after 1000 permutation analysis of seed oil content data across all genetic intervals. A total of 12 QTL including 6 QTL in Wuhan (OW), 4 QTL in Yangluo (OY), and 4 QTL in Qinghai (OQ) were detected in three trials using the software WinQTL Cartographer 2.5 (Table 4). These QTL were distributed over six linkage groups (A2, A3, A5, A6, C5, and C9) in Wuhan, four linkage groups (A2, A3, C8, and C9) in Yangluo, and four linkage groups (A2, A6, C2, and C9) in Qinghai.

**Table 4:** QTL analysis of seed oil content and gene markers in the QTL region in the DH population.

QTL^a^	QTL	QTL	Number^b^	Number^c^	LOD^d^	R^2^ e	ADD^f^	Source of	Gene^i^
	peak (cM)	region (cM)	(≤1 cM)	(≤5 cM)				increasing allele	marker
OW-A2	140.5	136.6–141.6	2	8	6.82	22.91	2.05	zy036	GSSR161
OW-A3	22.6	21.8–24.9	1	4	6.75	20.61	1.17^g^	zy036	
OW-A5	108.1	104.4–108.6	1	2	6.54	15.91	1.03	zy036	GIFLP106
OW-A6	47.2	46.3–50.4	2	3	3.85	13.15	−0.95^h^	51070	
OW-C5	72.6	72.4–76.2	0	1	4.95	15.82	1.6	zy036	
OW-C9	76.1	74.9–76.9	2	3	4.96	22.9	−1.58	51070	
OY-A2	26.3	20.8–37.5	1	6	2.96	9.15	0.35	zy036	GSNP39
OY-A3	29.1	27.6–29.5	3	7	7.11	24.56	0.77	zy036	
OY-C8	0	0–4.5	1	2	3.29	14.89	1.23	zy036	
OY-C9	29.9	27.1–33.1	2	7	3.71	11.79	0.36	zy036	GIFLP046
OQ-A2	140.5	138.3–143.5	2	8	2.61	21.75	1.23	zy036	GSSR161
OQ-A6	55.9	52.3–57.1	0	5	2.35	23.27	−1.19	51070	
OQ-C2	77.2	70.9–85	2	3	2.44	9.79	0.93	zy036	
OQ-C9	32	30.2–33.5	3	5	2.53	18.43	0.76	zy036	GIFLP046

a QTL related to seed oil content were named according to trials and corresponding linkage group number; e.g., *OW-A2* indicates QTL located in linkage group A2 in Wuhan trial (2010), *OY-A3* indicates QTL located in linkage group A3 in Yangluo trial (2011), *OQ-A6* indicates QTL located in linkage group A6 in Qinghai trial (2011).

b Number of linked markers ≤1 cM away from highest peak of QTL.

c Number of linked markers ≤5 cM away from highest peak of QTL.

d *LOD* value associated with detected QTL.

e Amount of phenotypic variation in total seed oil content (%) explained by a QTL.

f *ADD* additive effect (%) associated with detected QTL.

g Positive additive effect showing that zy036 alleles increased seed oil content expression compared with 51070 alleles at associated QTL.

h Negative additive effect showing that 51070 alleles increased seed oil content expression compared with zy036 alleles at associated QTL.

i Newly developed marker related to oil-content genes.

By QTL mapping, the amount of phenotypic variation (R^2^) in seed oil content explained by an individual QTL ranged from 9.15% (OY-A2) to 24.56% (OY-A3). This indicated that two genomic regions controlling seed oil content on the chromosome A2 (OW-A2 and OQ-A2) and C9 (OY-C9 and OQ-C9) in the DH population were co-localized in two out of three trials. The amount of phenotypic variation in seed oil content explained by QTL in the LG A2 was 22.91% (OW-A2) or 21.75% (OQ-A2), and that in the LG C9 was 11.79% (OY-C9) or 18.43% (OQ-C9). The other QTL were only be detected under one specific condition in the DH population.

The results from the QTL mapping in the DH population indicated that additive effects were the main factors contributing to variations in seed oil content. In the DH population, the additive effect of an individual QTL varied from 0.35% (OY-A2) to 2.05% (OW-A2). Analysis of the relative contributions of the alleles enhancing seed oil content in the two parents (zy036 and 51070) also revealed differences in the DH population. Among the 12 QTL identified in the DH population, zy036 contributed 9 seed oil content enhancing alleles (75.00%), while 51070 contributed 3 (25.00%). Interestingly, the QTL in the LGs A2 (OW-A2 and OQ-A2) and C9 (OY-C9 and OQ-C9), which were sources of alleles for increasing seed oil content, were from zy036. Therefore, those desirable alleles associated with enhancing seed oil content in these QTL regions may be derived from the specially high-oil parent zy036.

We found 34 linked markers in the 12 QTL regions(Table S4), 20 linked markers ≤1 cM and 50 linked markers ≤5 cM distant from the highest peak of the 12 QTL (Table 4). The number of linked markers in the QTL ranged from 1 (OW-A3, OW-A5, OW-C5, OW-C9 and OY-C8) to 7 (OW-A2), the number of linked markers ≤1 cM distant from the highest peak of the QTL ranged from 0 (OW-C5 and OQ-A6) to 3 (OQ-C9), and the number of linked markers ≤5 cM distant from the highest peak of the QTL ranged from 1 (OW-C5) to 8 (OW-A2 and OQ-A2).

To verify the correlations between 34 linked markers in the QTL and the seed oil content, we first used the method of single marker analysis by the software WinQTL Cartographer 2.5 (Table S4). The results showed all the markers in the QTL had significant correlations (P<0.05) with the seed oil content. Then, 34 linked markers were detected whether they showed significant distortion of segregation (P<0.01) using the chi-square test, and it was shown that 12 (33.33%) markers of 34 linked markers skewed to AA (zy036) genotype or BB (51070) genotype (Table S4). Finally, the significant difference analysis (P<0.05) in the mean seed oil content (± SE) between AA (zy036) genotype and BB (51070) genotype for every linked markers in the QTL was performed using T-test. All the 7 linked markers in two QTL in the LGs A2 (OW-A2 and OQ-A2) and C9 (OY-C9 and OQ-C9) had significant (P<0.05) or extremely significant (P<0.01) difference except GSSR161 in OQ-A2 and SF24724 in OY-C9 and OQ-C9. In addition, 17 markers (62.96%) out of the other 27 linked markers had significant or extremely significant difference. Details for significant differences of linked markers in the 12 QTL were summarized in Table S4. Such information will be useful for molecular marker assisted selection (MAS) breeding in the future.

### Analysis of Candidate Genes Associated with QTL

In the DH linkage map, there were 48 newly developed gene markers correlated with 47 differentially expressed genes. The detailed information about design, type, location, and sequence of 48 newly developed markers related to 47 differentially expressed genes in our DH map are summarized in Table S3. These 47 differentially expressed genes' functional roles were not only in the lipid synthesis, but also in sugar metabolism pathways, carbohydrate biosynthesis, and photosynthesis, unknown, or function as transcription factors that may be correlated with oil accumulation, and so on. By QTL mapping for seed oil content, we found four differentially expressed genes (*BnG44*, *BnG30*, *BnG40*, and *BnG36*). These four genes were associated with four gene markers (GSNP39, GSSR161, GIFLP106, and GIFLP046) in the QTL regions (Table 4), and were mapped in the following QTL: OY-A2, OW-A2/OQ-A2, OW-A5, and OY-C9/OQ-C9. These four differentially expressed genes may be candidate genes for seed oil content.

*BnG44*, a gene encoding the 10 kDa *PsbR* subunit of photosystem II (*PS*II), was associated with the gene marker GSNP39 in the QTL OY-A2. This subunit appears to be involved in the stable assembly of *PS*II, particularly that of the oxygen-evolving complex subunit *PsbP* [32]. *PS*II functions as a light-driven and water plastoquinone oxidoreductase, and its encoding genes are found in both nuclear and chloroplast genomes. This gene may be a candidate gene for seed oil content that is related to light.

*BnG30*, a gene associated with the gene marker GSSR161 in the QTL OW-A2/OQ-A2, is a member of the eight-member gene family encoding fatty acyl-CoA reductases (*FARs*)–*FAR1* [33]. Three of the *FARs* (*FAR1*, *FAR4*, and *FAR5*) generate fatty alcohols found in the root, seed coat, flower, and wounded leaf tissues [34], [35].

*BnG40*, a gene associated with the gene marker GIFLP106 in the QTL OW-A5, is one of three genes encoding subunit A of the trimeric protein ATP-citrate lyase (*ACL*)–*ACLA*. In plants, *ACL* is a heterooctamer consisting of *ACLA* and *ACLB* subunits. To function successfully, *ACL* enzymes must contain both subunits in their functional state [36]. Inhibition of activity of the A or B subunit can regulate the *ACL* gene. Fatland et al. [37] and Li et al. [28] validated that the *ACLA* gene was associated with seed oil content in *A. thaliana*. In our laboratory, we also found that the *ACLA* gene was correlated with seed oil content [38].

*BnG36*, a gene associated with the gene marker GIFLP046 in QTL OY-C9/OQ-C9, is a transcript that is differentially regulated at the level of mRNA stability at different times of the day, as controlled by the circadian clock [39]. This gene is affected by light intensity [40], and it may be a candidate gene for seed oil content.

**Figure 3:**
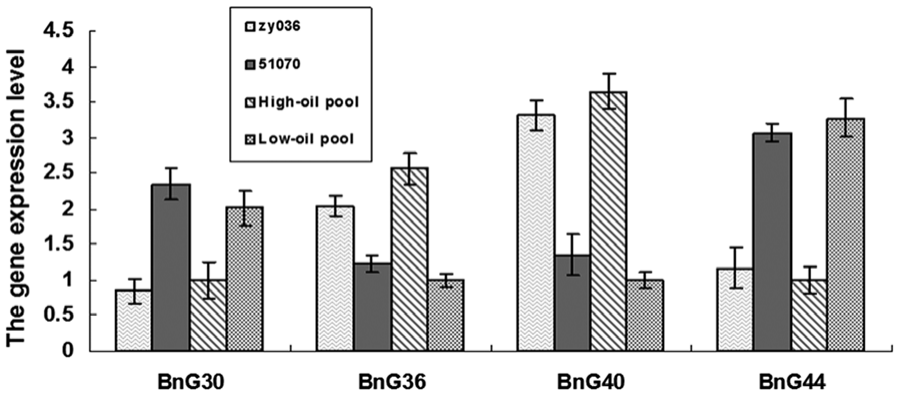
Real-time PCR analysis of the four candidate genes. Data expression was normalized to rapeseed *Bnactin*, and expression levels of four candidate genes (*BnG30*, *BnG36*, *BnG40* and *BnG44*) in rapeseed siliques (15 DAF) in zy036, 51070, and two pools of individuals in the DH population with high and low seed oil contents were compared with that of *Bnactin*. Data presented are mean values of three biological replicates, and error bars represent standard deviations.

To investigate these four genes expression pattern in detail, transcript levels were analyzed in rapeseed tissue siliques (15 days after flowering) in zy036, 51070, and two pools of individuals in the DH population with high and low seed oil contents. Real-time PCR results confirming differential expression of these four genes are summarized in Figure 3. The expression levels of these four genes had significant difference between parents and between two pools. Therefore, these four genes might be the promising candidate genes.

## Discussion

Improving seed oil content is an important research target for increasing the economic value of rapeseed. High-oil rapeseed cultivar can be used to find QTL associated with seed oil content, and those QTL associated with high oil content will be very useful for rapeseed breeding. In our experiments, we used the high oil content cultivar zy036 (seed oil content approximately 50%) and the low oil content cultivar 51070 (seed oil content approximately 36%). The parent zy036 is a specially high-oil cultivar which may contain excellent alleles for high oil content. In addition, there have been many studies on seed oil content in *B. napus* [12], [16], [21]–[24], [27]. In the above reported studies, the difference in seed oil content between the parents of the *B. napus* mapping populations ranged from 0.8 to 5.9%. The parents of our mapping population were high- and low-oil rapeseed cultivars showing more than 10% difference in their seed oil contents. To date, our experimental materials show the greatest difference between parents used to construct a *B. napus* mapping population. By using experimental materials with such large differences in seed oil content, we identified QTL that explain large amounts of phenotypic variation in seed oil content and that show large additive effects. In addition, we identified many differentially expressed genes associated with seed oil content.

By comparison with previous reported studies [7]–[20], IFLP primers in our experiments were the first time to be used in constructing genetic linkage map and mapping QTL in *B. napus*. In addition, SSR, SNP, and InDel had approximately equal amplified polymorphisms compared with those of previously reported primers [7]–[18]. As shown in Table 2, among the 5944 newly developed genome-sourced primers, amplified polymorphisms of SSR primers (593, 16.06%) in the parents were higher than those SNP primers (125, 5.55%). Among the 443 newly developed primers related to oil-content genes, amplified polymorphisms of SSR primers (41, 22.78%) or SNP primers (10, 23.81%) in the parents were higher than those IFLP primers (11,10.28%) or InDel primers (11, 9.65%). It showed that amplified polymorphisms of our genome-sourced SSR primers (16.06%) were approximately equal to those of publicly available SSR primers (17.83%) [7]–[14], [41], [42]. Amplified polymorphisms of our gene-sourced SSR primers (22.78%) were higher than those of publicly available SSR primers (17.83%) [7]–[14], [41], [42]. In addition, amplified polymorphisms of our gene-sourced SNP primers (23.81%) were higher than those of genome-sourced SNP primers (5.55%). These phenomena may be caused by the fact that gene-sourced SNP primers were developed based on cDNA sequences of differentially expressed genes in *B. napus*, while most of genome-sourced SNP primers (92.67%) were developed from *B. oleracea* resequencing.

Ecke et al. [21] constructed the first *B. napus* DH genetic linkage map to study QTL for seed oil content. Since then, more and more *B. napus* genetic linkage maps have been constructed for this purpose [12], [16], [22]–[24], [27]. These *B. napus* genetic linkage maps consisted of 214–481 markers, covering a total length of 1381–2690 cM with an average distance of 3.52–8.82 cM between markers. Using our DH population, we constructed a DH genetic linkage map using 527 molecular markers (Figure 2, Table 3) including 181 publicly available markers, 298 newly developed genome-sourced markers (Table S2) and 48 newly developed markers related to oil-content-genes (Table S3). The total length of the 19 linkage groups was 2,265.54 cM, with an average distance of 4.30 cM between markers. Compared with the most recently reported *B. napus* genetic linkage map, our map has more markers, greater total length, and shorter average distance between markers. As determined by chi-square tests, a total of 186 (35.29%) markers showed significant distortion of segregation (P<0.01) in our DH map, of which 64 markers skewed to zy036 and the other 122 skewed to 51070 (Table 3). Other *B. napus* DH mapping populations have also been reported to show significant deviations of 11.2, 35.7/20, 48, 55, 22, and 26.4%, respectively [12], [16], [21], [27], [43], [44]. Partial separation in the genetic process was the biological phenomenon observed in all of those studies, and is considered to be one of the biological evolutionary dynamics [45]. Partial separation is generated by gametophyte or sporophyte selection and discrepancies between the phenotype and genotype. In addition, markers linked to morphogenic and lethal loci are also prone to partial separation [46].

Here, we used a *B. napus* DH population derived from the cross between two cultivars showing a large difference in their seed oil content. This enabled the identification of QTL associated with large phenotypic differences in seed oil content. Also, there were additive effects of many markers linked to seed oil content. In the QTL analysis of our *B. napus* DH population, we found a total of 12 QTL distributed over seven linkage groups (A2, A3, A5, A6, C2, C5, C8, and C9). By comparison with previously reported studies [12], [16], [21]–[24], we had first mapped two QTL in LG C9. The amount of phenotypic variation in seed oil content explained by an individual QTL ranged from 9.15% (OY-A2) to 24.56% (OY-A3), and the additive effect of individual QTL ranged from 0.35% (OY-A2) to 2.05% (OW-A2) (Table 4). In various populations derived from different rapeseed cultivars, the positions of the detected QTL in the genome differed from those determined in this study [16], [22]. Based on the results of the genome sequence of *B. napus* (unpublished data), the scaffolds of the primer sequences were compared in the genetic linkage maps. Our seven QTL in LG A2 (OY-A2 and OW-A2/OQ-A2), A6(OW-A6 and OQ-A6), C5(OW-C5), and C9 (OY-C9/OQ-C9 and OW-C9) were new QTL compared with the QTL reported by Qiu et al. [22] and Zhao et al. [16]. Our QTL in LG C2 (OQ-C2) overlapped with the QTL in LG C2 reported by Qiu et al. [22], but differed from *OilC2* reported by Zhao et al. [16]. Our two QTL in LG A3 (OW-A3 and OY-A3) differed from the QTL in LG A3 reported by Qiu et al. [22], our QTL in LG A5 (OW-A5) differed from *OilC5* reported by Zhao et al. [16], and our QTL in LG C8 (OY-C8) differed from *OilC8-1* and *OilC8-2* [16]. Delourme et al. [12], Qiu et al. [22], Chen et al. [24], and Zhao et al. [16] reported for their own *B. napus* DH populations that the amount of phenotypic variation in seed oil content explained by individual QTL ranged from 1.2 to 13.4% and 4.6 to 19%, from 2.4 to 15.7%, from 4.20 to 30.20%, and from 2.85 to 26.50%, respectively, and that the additive effects of an individual QTL ranged from 0.28 to 1.02% and 0.36 to 0.73%, from 0.32 to 0.72%, from 0.47 to 1.53%, and from 0.27 to 0.89%, respectively. Compared with the *B. napus* QTL for seed oil content above, the values for the amount of phenotypic variation in seed oil content (R^2^) explained by an individual QTL and the additive effect of an individual QTL are higher in this study than those reported in most other studies. The alleles associated with increased seed oil content were mainly derived from the parent with high seed oil content. Of the 12 QTL associated with seed oil content identified in the present study, 9 were high-oil QTL which derived from specially high-oil cultivar zy036 (Table 4). There were 34 linked markers in the QTL (Table S4). These markers linked to the seed oil content in the QTL will be useful for MAS breeding.

Many studies have shown that the environment significantly affects phenotypic variation in rapeseed oil content in field trials and QTL for seed oil content [12], [16], [22]–[24]. To obtain more reliable and stable QTL for seed oil content, we conducted field trials in three different environments. There were strong environmental effects on average seed oil content in the DH population (Figure 1B). The seed oil content in the DH population in the Wuhan trial (2010) was lower than that in the Yangluo (2011) and Qinghai (2011) trials. Delourme et al. [12] reported similar results: their DH *B. napus* population designated as DY showed a lower seed oil content in the SE02 trial (38–48%) than in the RE01 (38–54%) and RE02 trials (43–52%), and the DH population designated as RNSL showed a lower seed oil content in the ROSEN trial (35.7–47.7%) than in the CVIL (38–48.2%) and VERN trials (36.6–50.0%). The environment greatly affected the stability of the QTL over the different trials in our DH population. Among the 12 QTL for seed oil content, 2 QTL were consistent in two out of three trials in our DH population. The amount of phenotypic variation explained by the two high-oil QTL in LG A2 was approximately equal (22.91% for OW-A2, 21.75% for OQ-A2), while in LG C9, OY-C9 explained 11.79% and OQ-C9 explained 18.43% of the phenotypic variation (Table 4). Zhao et al. [16] showed that the major QTL OilA7 was significantly observed in all 11 experiments, explaining 4.97 to 26.50% of phenotypic variation in the *B. napus* DH population, while the other eight QTL were only detected in one or several regions. These phenomena may be because some genomic regions controlling seed oil content are common in different genetic backgrounds, while some are not. Furthermore, in every segregating population, the parents may have the potential to reveal genetic limiting factors.

The great advantage of our experiment is that gene markers correlated with seed oil content were used to construct the genetic linkage map. These gene markers, which came from differentially expressed genes related to seed oil content in *B. napus*, were mapped in the genetic linkage map. This enabled screening and identification of candidate genes for seed oil content. Molecular genetics and molecular biology studies have shown that there are three main types of genes affecting seed oil content in plants. First, there are hundreds of genes encoding dozens of enzymes that control the lipid biosynthesis pathway [47] and altering expression of a key gene can change seed oil content [48]; second, there are a number of genes that encode key enzymes (e.g., hexokinase, pyruvate kinase, and glucose-6-phosphate dehydrogenase) affecting the distribution of carbon to starch, oil, and protein [49]; third, there are sets of key transcription factors such as *LEC1*, *LEC2*, *FUS3*, and *WRI1* [50]. Some studies have screened candidate genes for seed oil content by detecting whether orthologous genes are related to lipid biosynthesis pathway in *A. thaliana* mapped to QTL regions [16], [27]. In our study, we chose a few types of differentially expressed genes which might affect seed oil content in plants. These genes likely have functions in the lipid synthesis, sugar metabolism pathways, carbohydrate biosynthesis, and photosynthesis, unknown function, or function as transcription factors that may be correlated with oil accumulation, and so on. In the present QTL analysis for seed oil content, we identified four candidate genes related to four gene markers: GSNP39, GSSR161, GIFLP106, and GIFLP046. Furthermore, the expression levels of these four genes had obvious difference between parents and between two pools (Figure 3). Therefore, these four genes might be the promising candidate genes.

## Conclusions

In the present study, we demonstrated that it would be very useful for rapeseed breeding to find those QTL associated with high oil content. There are now high-oil alleles among the high-oil rapeseed cultivars in nature. Our experimental results show that it is important to study the specially high-oil *B. napus* cultivar zy036. And analysis of special rapeseed cultivar with high oil content enabled us to map high-oil QTL. In addition, to address the lack of polymorphic markers, we designed more than 6000 pairs of new primers based on *B. rapa*, *B. oleracea*, and *B. napus* genome sequence information or *B. napus* differentially expressed genes associated with oil content. To identify high-oil QTL, we constructed a new DH genetic linkage map which had more markers, greater total length, and shorter average distance between markers compared with the most recently reported *B. napus* genetic linkage map. Further, we found many linked markers in our trials. These markers linked to the seed oil content in the QTL will be useful for MAS breeding. We also found 4 candidate genes that were *B. napus* differentially expressed genes. These genes are likely related to regulation of oil content. Cloning those genes related to high oil content provides greater possibilities to improve the seed oil content of rapeseed in the future.

## Materials and Methods

### Ethics Statement

No specific permits were required for the described field studies. No specific permissions were required for these locations/activities. The location is not privately-owned or protected in any way. The field studies did not involve endangered or protected species.

### Plant Materials

The *B. napus* doubled haploid (DH) lines were produced from F_1_ plants following microspore culture protocols. Microspores were isolated and cultured according to the protocol described by Wang et al. [51], [52]. The F_1_ hybrids were derived from a cross between the *B. napus* cultivars zy036 and 51070. The parent zy036 is a specially high-oil cultivar whose seed oil content is approximately 50% and the parent 51070 is a specially low-oil cultivar whose seed oil content is approximately 36% [29], [30]. The obtained F_1_ plants were used to develop a population of 112 DH lines. For convenience, we randomly selected a subset of 92 lines from the DH population for field trials, seed oil content measurement, linkage map construction, and QTL mapping for seed oil content.

### Field Trials and Seed Oil Content Measurement

The field experiments with the high oil content parent zy036, low oil content parent 51070, and the DH population were conducted in a randomized complete block design with three replicates. The seeds were sown by hand in double rows in each plot, and the field management followed standard agricultural practices. Each row was 2.5 m long, with 40 cm between rows and 20 cm between individual plants. At maturity, six to ten individual rapeseed plants were harvested from each plot in Wuhan (2010), Yangluo (2011), and Qinghai (2011), in China. The seed oil content was estimated using a Foss NIRSystems 5000 near-infrared reflectance spectroscope according to the WinISI III manual instructions for routine analysis (Foss NIRSystems Inc, http://www.foss-nirsystems.com).

### Design of Primers

The complete *Brassica* A genome sequence from *B. rapa*, derived from de novo assembly of sequence scaffolds using second-generation sequencing technologies, has been published online http://brassicadb.org/brad/ [53]. The entire genome sequences of *B. oleracea* and *B. napus* have also been determined (unpublished data). Based on the results of the genome sequences of *B. rapa*, *B. oleracea*, and *B. napus*, we designed genome-sourced primers to amplify SSRs and SNPs in our laboratory.

Using the method of bulked segregant analysis (BSA), extreme genotypes for a quantitative trait were collected and the RNA from contrasting bulks was then profiled with the aim of finding differentially expressed genes[54], [55]. In addition, the high-/low-expression of genes was one of the most important factors affecting the major QTL involved in agronomic traits [56]. These methods are valuable because they combine the advantage of gene expression and BSA which can rapidly find differentially expressed genes or identify markers by two bulked RNA samples. In our studies, a total of two pools (one consisting of individuals with extremely high oil content and one consisting of individuals with extremely low oil content) were analyzed at the transcriptome level using Solexa/Illumina sequencing, and many differentially expressed genes were found (unpublished data). These two pools with 10 lines per pool were assembled using siliques of 15 days after flowering (DAF) in our DH population. Two pools' RNAs were isolated from 15 DAF siliques which were mixed equivalently using a Plant Mini RNeasy kit (Qiagen; http://www.qiagen.com/) and Solexa/Illumina sequencing was carried out by BGI Shenzhen, China. We developed new primers according to the sequences of the differentially expressed genes. The newly developed primers were related to oil-content genes and amplified SSR, IFLP, SNP, and InDel markers.

### Publicly Available Primers

The 2244 pairs of publicly available primers were all SSR primers. The following prefixes indicate the origin of these SSR primers: “BN” were developed from the *B. napus* genomic sequence [7], [8]; “BnEMS”, “BnGMS”, “BoGMS”, and “BrGMS” were provided by Huazhong Agricultural University, China; “BRAS” and “CB” were from Piquemal et al. [11] and Radoev et al. [13]; “CUN” were provided by the Chungnam National University, Korea; “EJU” and “ENA” were from Choi et al. [41]; “FITO” were developed by Iniguez-Luy et al. [14]; “IGF” were provided by the *Brassica* IGF Project; “MR” were developed by Uzanova and Ecke [9]; “Na”, “Ni”, “Ol” and “Ra” were developed by Lowe et al. [10], [42]; “niab” were provided by the National Institute of Agricultural Biotechnology, Korea; and “sN”, “sR” and “sS” were provided by Agriculture and Agri-Food, Canada.

### Newly Developed Genome-Sourced Primers

We designed 5944 pairs of genome-sourced primers including those that amplified SSRs and SNPs. The 3693 pairs of SSR primers prefixed by “BrSF” were developed from *B. rapa* scaffold sequences, those prefixed by “BrBAC” and “P” were developed from *B. rapa* BAC sequences, and those prefixed by “BoSF” and “SF” were developed from *B. oleracea* scaffold sequences. The 2251 pairs of SNP primers prefixed by “snap” were developed from *B. oleracea* resequencing, those prefixed by “pr” were developed from *B. napus* scaffold sequences, and those prefixed by “ns” were developed from *B. napus* resequencing.

### Newly Developed Primers Related to Oil-Content genes

Based on the results of the genome sequences of *B. napus* (unpublished data), we obtain the sequence of all annotation genes (over 80,000), and can distinguish each paralogous *B. napus* gene. We chose 50 differentially expressed genes (Table S1) to design newly developed primers related to oil-content genes. These primers were used to amplify SSR, IFLP, SNP, and InDel markers.

1. Based on the results of the genome sequences of *B. napus*, simple sequence repeats round differentially expressed genes in the *B. napus* scaffolds could be found. We developed 180 pairs of SSR primers (prefixed by “GSSR”) in the both sides of flanking sequences which were ≤10 Kb distant from these differentially expressed genes.
2. By comparison between cDNA sequences of *B. napus* differentially expressed genes and DNA sequences of *B. napus* genome, possible introns and exons were showed. By crossing the intron sequences of *B. napus* genes, IFLP primers were designed to amplify exon sequences. In total, we developed 107 pairs of IFLP primers (prefixed by “GIFLP”);
3. Mutation-deletion oligonucleotides were developed based on cDNA sequences of differentially expressed genes in *B. napus*. To reduce the probability of amplifying multiple fragments, most primers were amplified exon sequences of genes. Totally, we developed 42 pairs of SNP primers (prefixed by “GSNP”);
4. We analyzed the cDNA sequences of *B. napus* genes that were differentially expressed genes between two pools of individuals in the DH population with high and low seed oil contents, and found many inserted-deleted oligonucleotides in the cDNA sequences. The InDel markers were designed to detect three sequential insertion-deletion oligonucleotides in close proximity. In all, we developed 114 pairs of InDel primers prefixed by “Gindel” and “GTFzip”.

All of the primers were designed using the software Primer3 [57]. The primers for amplifying SSR, IFLP and InDel markers ranged from 18 to 27 nucleotides in length. The newly developed SNP primers related to oil-content genes length ranged from 18 to 20 nucleotides in length and the newly developed genome-sourced primers for amplifying SNPs ranged from 21 to 36 nucleotides in length. The melting temperatures ranged from 50 to 70°C. The optimum temperature for SSR, IFLP, InDel, and the newly developed SNP primers related to oil-content genes was 55°C and that for the newly developed genome-source SNP primers was 62°C. The optimum GC content was set to 50%, with a minimum of 30% and a maximum of 70%. The predicted PCR products ranged from 100 to 500 bp.

### Amplification of Primers

For primer screening and genotyping, DNA was isolated from zy036, 51070, and the DH population using the cetyltrimethylammonium bromide (CTAB) DNA extraction method [58]. The quality and concentration of DNA was determined using a Nanodrop ND1000 spectrophotometer. The DNA was diluted to 50 ng/µl.

PCR reactions using primers to amplify SSR, IFLP, SNP, and InDel markers from the parents and DH population were performed in 96-well plates. Each reaction mixture contained 1 U Taq DNA polymerase, 2 mM MgCl_2_ in 1×buffer, 200 µM dNTP, 0.2 µM forward primer, 0.2 µM reverse primer, 50 ng DNA, and ddH_2_O to complete the volume to 20 µl.

The PCR using SSR primers was performed using the following program: 94°C for 4 min; 10 cycles 94°C for 30 s, annealing temperature for 30 s, 72°C for 1 min, with an annealing temperature starting at 60°C and decreasing by 0.5°C each cycle; then, 30 cycles of 94°C for 30 s, 55°C for 30 s, and 72°C for 1 min; and then a final elongation step of 10 min. Finally, PCR amplification products were separated by 6% denaturing polyacrylamide gel electrophoresis (PAGE) and visualized by silver nitrate staining.

The PCRs using IFLP, SNP, and InDel primers were performed with the following program: 94°C for 4 min; 40 cycles with 94°C for 30 s, primer-specific annealing temperature for 30 s and 72°C for 1 min; and then a final elongation step of 10 min. Finally, PCR amplification products were separated by nondenaturing PAGE on 8% acrylamide gels and visualized by silver nitrate staining.

### Construction of Genetic Linkage Map

The genetic linkage map was constructed using Joinmap 3.0 [59]. All genetic distances are expressed in centimorgan (cM) units using the Kosambi function [60]. The threshold for goodness of fit was set to ≤5.0 with logarithm of the odds ratio (LOD) scores of >1.0 and a recombination frequency <0.4. The segregation of each marker in the DH population was analyzed by a chi-square test for “goodness-of-fit” with a ratio of 1∶1. The linkage groups are believed to map to the same chromosome as predicted from public markers used as anchor markers.

### QTL Mapping for Seed Oil Content

QTL mapping of seed oil content in the DH population was performed by composite interval mapping (CIM) [61], [62] using the software WinQTL Cartographer 2.5 [63]. The statistical significance of each QTL was determined by its LOD score and the percentage of seed oil content variation that it explained [64], [65]. Statistical levels for LOD scores were determined by 1000 permutation analysis of seed oil content data across all genetic intervals. The likelihood ratio test (LRT) thresholds were estimated at 16.3 in Wuhan (2010), 13.5 in Yangluo (2011), and 9.4 in Qinghai (2011).

### Real-Time PCR

Using the Plant Mini RNeasy kit (Qiagen), the four RNAs of zy36, 51070, and two pools of individuals in the DH population with high and low seed oil contents were extracted from their tissues (siliques of 15 DAF). The reverse transcription reaction was performed using the First Strand cDNA Synthesis Kit for Real-time PCR (Takara, Dalian, China). Primers (BnG30RTF/R, BnG36RTF/R, BnG40RTF/R and BnG44RTF/R) were designed to detect expression of *BnG30*, *BnG36*, *BnG40*, and *BnG44* in rapeseed. *Bnactin* served as endogenous reference genes. The real-time PCR contained 1 µl of 10-fold diluted first-strand cDNA, 1 µl of SYBR Green (Applied Biosystems, Carlsbad, CA, USA), 0.2 mM dNTP, 1× LA buffer, 0.5 U of LA Taq (Takara) and 10 µM of each primer. Initial denaturation time was 10 min, followed by 40 cycles of 95°C for 15 s, 55°C for 30 s, and 72°C for 30 s. A melting curve was performed after PCR cycles to verify that only one PCR product was amplified. Experiments were performed in triplicate and data were obtained from three independent experiments with similar results. The absolute slope values of the curve of log cDNA dilution versus DC(T) were assessed and the efficiencies of primers for target and reference genes were considered to be equal if the absolute slopes were <0.1. Primers used for real-time PCR are listed in Table S5.

## Supporting Information

Table S1 Details for 50 differentially expressed genes between two pools of individuals in the DH population with high and low seed oil contents.
(XLS)

Table S2 Details for 298 newly developed genome-sourced markers in DH genetic linkage map.
(XLS)

Table S3 Details for 48 newly developed markers related to oil-content genes in DH genetic linkage map.
(XLS)

Table S4 Details for correlations between linked markers in the QTL and the seed oil content.
(XLS)

Table S5 Details for primers used for real-time PCR of four candidate genes.
(XLS)
